# Metabolite Profiling and Quantitation of Cucurbitacins in Cucurbitaceae Plants by Liquid Chromatography coupled to Tandem Mass Spectrometry

**DOI:** 10.1038/s41598-019-52404-1

**Published:** 2019-11-05

**Authors:** Faraz Ul Haq, Arslan Ali, Muhammad Noman Khan, Syed Muhammad Zaki Shah, Ram Chandra Kandel, Nudrat Aziz, Achyut Adhikari, M. Iqbal Choudhary, Atta- ur-Rahman, Hesham R. El-Seedi, Syed Ghulam Musharraf

**Affiliations:** 1 H.E.J. Research Institute of Chemistry, International Center for Chemical and Biological Sciences, University of Karachi, Karachi, 75270 Pakistan; 2 Dr. Panjwani Center for Molecular Medicine and Drug Research, International Center for Chemical and Biological Sciences, University of Karachi, Karachi, 75270 Pakistan; 3 Central Department of Chemistry, Tribhuvan University, Kirtipur, Kathmandu, Nepal; 4 Department of Biochemistry, Faculty of Science, King Abdulaziz University, Jeddah, 21452 Saudi Arabia; 5 Pharmacognosoy Group, Department of Medicinal Chemistry, Uppsala University, Biomedical Centre, Box 574, SE-75 123 Uppsala, Sweden; 6 Alrayan Medical College, Medina, 42541 Kingdom of Saudi Arabia

**Keywords:** Mass spectrometry, Cheminformatics

## Abstract

Cucurbitaceae is an important plant family because many of its species are consumed as food, and used in herbal medicines, cosmetics, etc. It comprises annual vines and is rich in various bioactive principles which include the cucurbitacins. These steroidal natural products, derived from the triterpene cucurbitane, are mainly the bitter principles of the family Cucurbitaceae. Their biological activities include anti-inflammatory, hepatoprotective, and anti-cancer activities. A total of 10 species belonging to 6 genera of the Cucurbitaceae family along with *Cissampelos pareira* (Menispermaceae) were included in this study. A comprehensive profiling of certain natural products was developed using HPLC-QTOF-MS/MS analysis and a distribution profile of several major natural products in this family was obtained. A total of 51 natural products were detected in both positive and negative ionization modes, based on accurate masses and fragmentation patterns. Along with this, quantitation of four bioactive cucurbitacins, found in various important plants of the Cucurbitaceae family, was carried out using multiple reaction monitoring (MRM) approach on an ion trap mass spectrometer. Cucurbitacin Q was found to be the most abundant in *C. pareira*, while *Citrullus colocynthis* contained all four cucurbitacins in abundant quantities. The developed quantitation method is simple, rapid, and reproducible.

## Introduction

Cucurbitacins are steroids derived from the triterpene skeleton “cucurbitane”. They are well distributed in plants of the Cucurbitaceae family. Although cucurbitacins were originally isolated as bitter principles from plants of the Cucurbitaceae family, they are now known to occur in other plant families, such as Brassicaceae, Scrophulariaceae, Begoniaceae, Elaeocarpaceae, Datiscaceae, Desfontainiaceae, Polemoniaceae, Primulaceae, Rubiaceae, Sterculiaceae, Rosaceae, and Thymelaeaceae^1^. Occurring as either glycosylated or non-glycosylated molecules, these compounds are well known for their toxicity and biological activities, such as cytotoxicity^2–4^, anti-inflammatory activity^5^, anti-malarial activity^6^, hepatoprotective potential^7^, and other activities.

The family Cucurbitaceae, also known as the gourd family, consists of 965 plant species in 95 genera^8^, which mostly occur as annual vines. Several of these are consumed as vegetables and fruits. Most commonly consumed plants in this family are various pumpkins, gourds, calabash, cucumber, melon, and watermelon varieties. The literature in this field is expanding and various research groups have reported biologically active cucurbitacins from several plants such as *Citrullus colocynthis*, *Momordica charantia* and others^9–13^. Since cucurbitacins have various interesting biological activities and are widely distributed among the plants of Cucurbitaceae family, there is a need to establish metabolite distributions of important genera and species in this family.

Metabolic profile development requires prior identification of natural products for which HPLC-MS/MS is a fast and reliable method. It is often done without the use of chemically pure standards as the availability of a compound in question through synthesis, isolation or commercial sources is not always possible. However, the level of certainty in natural product identification through mass spectrometry varies. This depends upon whether the data was obtained in reference to purified standards or only an untargeted study was performed. Authentic identification through mass spectrometry is only possible when a purified compound is available. However, in case of a metabolomics study it is neither economical nor practically possible to have a large number of purified standards available. However, in cases where purified standards are not available, it is still possible to identify natural products through a sample based on MS/MS fragmentation data^14^.

Keeping in view the important bioactivities of cucurbitacins, quantitation of these compounds in various plants of the Cucurbitaceae family was carried out. Various reports on the quantitation of important cucurbitacins in plants such as the zucchini^15^, bottle gourd^16^ and bitter melon^17^ have appeared in the literature. A notable report is the quantitation and pharmacokinetics study of cucurbitacin IIa and cucurbitacin IIb from *Hemsleya amabilis* in rat plasma. These two compounds are considered major bioactive constituents in this plant and have promising antiproliferative activities^18^.

We present herein a comprehensive study focusing on the generation of metabolic profile of various plants of the Cucurbitaceae family, along with quantitation of four biologically active cucurbitacins in ten species belonging to six different genera of the Cucurbitaceae family, along with *Cissampelos pareira* belonging to the Menispermaceae family. *C. pareira* is also reported to possess antioxidant^19^, anti-inflammatory^20^, antiviral^21^, antidiabetic^22^, anticancer^23,24^ and other activities^25^. There are studies in literature that report of the profiling of Cucurbitaceae family plants, such as profiling of phenolics and other polar components in watermelon^26^ and zucchini^27^ through LC-MS/MS. The study presented herein should serve as a stepping stone for more detailed metabolomics studies on important plants of the Cucurbitaceae family.

## Experimental

### Chemicals and reagents

**Figure 1:**
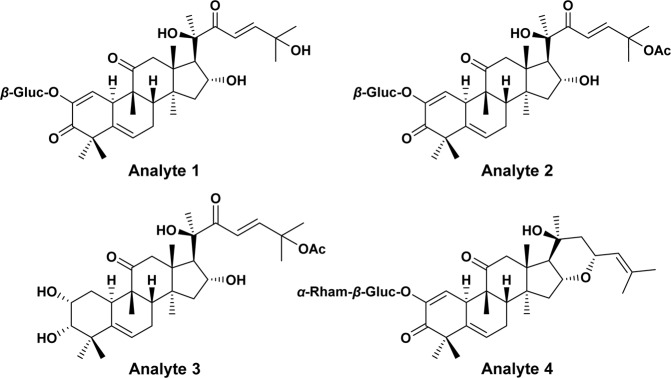
Chemical structures of analytes **1**–**4** quantitated in various plants.

Analytes **1**, **2** and **4** namely cucurbitacin E 2-*O*-*β*-D-glucopyranoside (**1**), cucurbitacin I 2-*O*-*β*-D-glucopyranoside (**2**) and 22-deoxocucurbitoside B (**4**) were isolated from methanolic extract of *Citrullus colocynthis* fruits. The crude methanolic extract was fractionated using dichloromethane (DCM) and ethyl acetate. Compound **1** was isolated from the DCM fraction, while compounds **2**–**4** were isolated from the ethyl acetate fraction. Cucurbitacin (**3**) was isolated from DCM extract of *C. pareira*. The DCM extract was fractioned using hexanes and ethyl acetate. The structures of analytes are shown in Fig. 1. The compounds were characterized based on comparison of their ^1^H- and ^13^C-NMR spectral data with the data reported in literature^28,29^. Details of isolation are provided with supplementary information along with necessary spectroscopic data.

Formic acid, purchased from Daejung (Daejung Chemicals & Metals Co. Ltd., Korea), was used as an additive for the mobile phase. Methanol for mobile phase was purchased from Merck (Merck KGaA, Darmstadt, Germany). Type I water (ISO 3696) for the mobile phase was obtained from a Barnstead™ GenPure™ ultrapure water system (Thermo Fisher Scientific Inc., USA).

### Instrumentation and experimental conditions

HPLC-ESI-MS/MS analysis for natural product identification was performed on a Bruker maXis II™ HR-QTOF mass spectrometer (Bremen, Germany), coupled to a Dionex UltiMate™ 3000 series HPLC system (Thermo Fisher Scientific, Waltham, MA, USA) fitted with a binary RS pump, column thermostat, and auto-sampler. Sample chromatography was performed on a Macherey-Nagel Nucleodur® C18 Gravity column (3.0 × 100 mm, 1.8 µm), kept at 40 °C. 4-µL samples were injected while the mobile phase consisted of A (0.1% formic acid in H_2_O) and B (0.1% formic acid in MeOH). The mobile phase flow rate was set at 0.7 mL/min using a linear gradient of A and B starting at 10% B, increased to 90% B in 5.5 min, maintained at 90% for 1.5 min, and returned to 10% B in 1 min. The total run time was 10 min, including a 1 min holding time at the start and 1 min equilibration time at the end of the gradient.

Mass spectra were recorded using electrospray ionization employing the Bruker CaptiveSpray™ ion source. MS and MS/MS spectra were recorded separately both in positive and negative modes. Ion source parameters were set as follows (parameters for negative mode next to positive mode parameters): capillary voltage at 4500 V (−3500 V), end plate offset at 500 V, nebulizer gas 45.0 psi, drying gas at 12.0 L/min and drying gas temperature at 270 °C. All spectra were recorded in the mass range of *m/z* 100 to 2000, while the scan speed was set at 5 Hz for MS and 12 Hz for MS/MS spectra. Active exclusion feature of the instrument was used which enables the instrument to remove a precursor ion from further consideration after a set number of MS/MS spectra have been recorded for that particular precursor ion. The active exclusion number was set at 3, and the precursor reconsideration time was set at 30 s.

HPLC-MS/MS analysis for quantitation was performed on a Bruker amaZon speed ion trap mass spectrometer (Bremen, Germany), coupled to a Dionex UltiMate™ 3000 series HPLC system (Thermo Fisher Scientific, Waltham, MA, USA) fitted with a binary pump, column thermostat and auto-sampler. Chromatographic separation of analytes was achieved on a reverse-phase C18 column (Agilent Poroshell 120 EC-C18 3.0 × 50 mm, 2.7 µm), kept at 40 °C. 2-µL samples were injected while the flow rate was set at 0.5 mL/min. A linear gradient was used for analyte elution starting at 10% B, increased to 95% B in 3.5 min, maintained at 95% for 1.5 min, and returned to 10% B in 1 min. The column was equilibrated for 1 min at the end of the gradient. Total run time for analysis was 8 min.

Mass spectra were recorded using electrospray ionization under positive mode employing the Bruker CaptiveSpray™ ion source. Ion source parameters were set as follows: capillary voltage at 4500 V, end plate offset at 500 V, nebulizer gas 35.0 psi, drying gas at 8.0 L/min, and drying gas temperature at 250 °C. Mass spectra scan range was set at *m/z* 100 to 1000, while the number of spectral averages was set at 3. Ion charge control (ICC) was used for transferring a certain number of ions to the ion trap and set at 60,000, while accumulation time was set at 100 ms. Fragmentation time under collision-induced dissociation (CID) mode was set at 20 ms while fragmentation amplitude was optimized for each analyte to obtain the maximum abundance of fragment ions.

### Method performance

All MS and MS/MS data were saved using both profile and line spectra to minimize the chance of instrumental noise being taken as a precursor ion. Mass spectra for all samples were recorded under both ionization modes (positive and negative) to counter check the authenticity of a molecular ion peak while active exclusion was used to minimize the chances of common contaminant peaks being placed under MS/MS fragmentation. Each sample was injected in triplicate.

The developed quantitation method was assessed for accuracy and precision. Accuracy (% bias) and precision (% RSD) were assessed by analyzing three different QC samples with six replicates for intra-day, and 12 replicates on two different days (6 replicates/day) for inter-day analysis. Excellent accuracy and precision (<5%) were found for the developed method. The accuracy of analysis was calculated using the expected concentration (*C*_E_) and the mean value of measured concentration (*C*_M_) by using the following relation: Accuracy (bias, %) = [(C_E_-C_M_)/C_E_]x100. Similarly, the relative standard deviation (% RSD) was used as an indicator of the analytical precision, and calculated from the standard deviation and mean value of measured concentrations by the following equation: Precision (RSD, %) = (Standard Deviation (SD)/C_M_)x100. Method performance was further evaluated through the analysis of fortified samples prepared by spiking additional amounts of analytes **1–4** at three levels of 50, 100, and 150 ng/mL, respectively, in the original sample solutions used for analysis. Details about method precision and validation along with calibration equations, LOD and LOQ values are provided with supplementary information (Supplementary Tables 1, 2 and 3).

### Sample preparation

Shade-dried plant material (whole plants) were crushed in a blender. 1 g of each plant was weighed and extracted with 10 mL methanol through sonication for 20 min. Each sample was centrifuged for 15 min at 6000 rpm to settle large particles, and the supernatant was filtered through a 0.22 µm PTFE syringe-driven filter. 50 µL of the filtered extract was diluted to 1000 µL with methanol for LC-MS, and LC-MS/MS analysis.

For quantitation, 1 mg of each standard compound was weighed and dissolved into 1 mL methanol to prepare standard stock solutions. These solutions were diluted with 50:50 water: methanol in a serial manner to prepare ten calibrant solutions ranging between 50–2000 ng/mL. The analysis of plant samples was performed using diluted plant extract. 50 µL of filtered plant extract was diluted to 1500 µL with 50:50 water: methanol for LC-MS/MS analysis.

Spiked samples for method validation were prepared in a similar manner as the plant samples. 50 µL of filtered plant extract plus an amount of standard solution equivalent to spike concentrations of 50, 100, and 150 ng/mL was diluted to a final volume of 1500 µL with 50:50 water: methanol for three samples, and labelled as S1, S2 and S3, respectively.

## Results and Discussion

### LC-MS/MS optimization

The method for LC-MS/MS in the profiling study was optimized using a RP-C18 column in a way that the various sample components eluted in a 10 min runtime. No carryover was detected in the next blank sample run after the plant sample. Analysis were performed in both positive and negative ionization modes, and the mobile phase composition for both polarities was kept identical (0.1% formic acid in both solvents). However, to obtain a reasonable cycle time for the MS/MS analysis, the scan frequency of the instrument was kept at maximum (12 Hz), and active exclusion was used to avoid solvent contaminant peaks being placed under MS/MS fragmentation. Precursor reconsideration time was set at 0.5 min after careful examination of the peak widths. This ensured that no precursor ions are excluded from the analysis.

**Figure 2:**
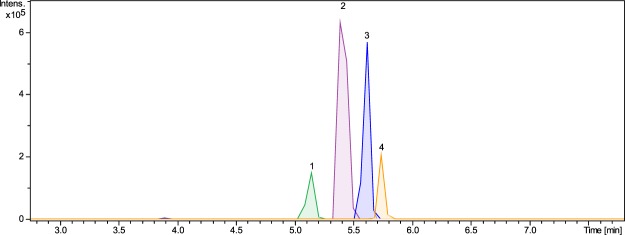
Extracted-ion chromatogram of standard analytes **1**–**4** analyzed by MRM.

The mobile phase gradient for quantitative analysis was adjusted in order to elute all analytes in the shortest possible runtime and to have large enough differences in runtimes to avoid overlapping MRM transitions. Figure 2 shows that all analytes eluted from the column between 5.0–6.6 min of analysis with small peak widths, and without any observable peak tailing or fronting. The analysis was performed under positive ionization mode, and all of the analytes were observed as sodium adducts ([M + Na]^+^). Due to the large structures and presence of various oxygen atoms (as hydroxyls) in the structures, all analytes showed a good affinity towards the formation of sodium adducts. Ammonium adducts were also observed along with sodium adducts when the mobile phase composition was changed from 0.1% formic acid to 20 mM ammonium acetate. However, the use of ammonium acetate decreased the instrument sensitivity. 0.5% acetic acid was also used as a mobile phase, but this resulted in a lower sensitivity as compared to 0.1% formic acid in positive mode, whereas chloride adducts were obtained in negative mode. 0.1% formic acid in negative mode also resulted into the formation of formate adducts. However, it was observed that all analytes in negative mode with different mobile phase compositions showed smaller instrumental response as compared to sodium adducts in the positive mode. Therefore, it was concluded that 0.1% formic acid in positive mode was the best mobile phase for analysis.

**Figure 3:**
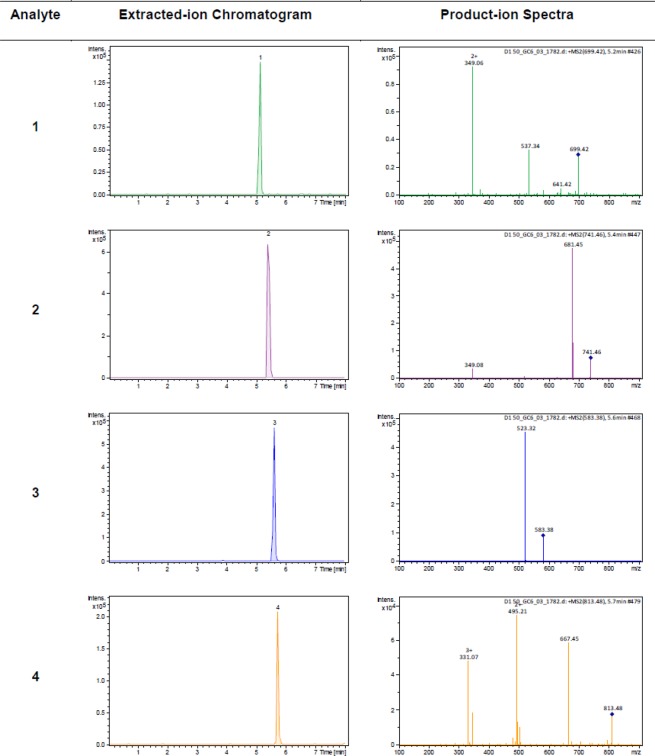
Representative MRM extracted ion chromatograms and product ion spectra of standard analytes **1**–**4** at 50 ng/mL.

The observed sodium adducts were subjected to MS/MS fragmentation analysis in the ion trap and the fragmentation amplitudes were tuned for each analyte. All analytes showed good fragment yields in the fragmentation amplitude range of 0.90–1.55 V. Table 1 summarizes optimized MRM parameters for analytes **1**–**4**. A standard mixture of analytes was prepared at a concentration of 50 ng/mL and analyzed under optimized chromatographic and MRM conditions. Excellent chromatographic peak shapes with good intensities were observed (Fig. 2). Figure 3 shows extracted ion-chromatograms, and product ion spectra of analytes **1**–**4** in the standard mixture at a concentration of 50 ng/mL.

**Table 1:** Optimized MS/MS parameters for analytes **1**–**4**.

Analyte	Compound analyzed	Retention time (min)	[M + Na]^+^ (*m/z*)	Fragmentation amplitude (V)	MRM transitions (*m/z*)
1	Cucurbitacin I 2-*O*-*β*-D-glucopyranoside	5.15	699.4	1.30	699.4→349.1, 537.4
2	Cucurbitacin I 2-*O*-*β*-D-glucopyranoside	5.44	741.4	1.10	741.4→681.4, 349.1
3	Cucurbitacin Q	5.61	583.4	0.90	583.4→523.4
4	22-Deoxocucurbitoside B	5.72	813.4	1.55	813.4→495.2, 667.4, 331.1

### Identification and quantitation of natural products

A total of 51 compounds were putatively identified based on their high-resolution masses and fragmentation data in positive and negative ionization modes. Their identification was performed using a library of natural products previously reported from the plant species included in this study. The library was custom-built as follows. Plant names (with all synonyms) were queried in the Dictionary of Natural Products (DNP) Ver. 26.2 (Dec 2017), and all resulting hits were used to build a library of natural products. All the samples were screened against the prepared library using Bruker Compass TargetAnalysis Ver. 1.3 software, which compares the mass errors (ppm) and isotopic patterns of the compounds in the library with the observed mass spectra and ranks the probable compounds based on match score. The samples were then analyzed again for MS/MS spectra of compounds which were found using TargetAnalysis. Entries with higher ppm errors (>10 ppm) were discarded, and no MS/MS data analysis was performed. It was observed that mass errors were below 2 ppm in most cases. The fragment ions in the MS/MS data were analyzed using *in-silico* fragmentation. Fragments were generated by manually dissecting the molecules at various possible sites and comparing the theoretical fragments with those obtained from the data. Details about the compounds identified in positive and negative ionization modes are presented in Table 2.

**Table 2:** Table of compounds detected in positive and negative ionization modes.

S. No.	Name	Molecular Formula	RT (min)	m/z	Ion Type	Exact Mass	error (ppm)	MS/MS
1	2,16,19,20,25-Pentahydroxycucurbit-5-ene-3,11,22-trione	C_30_H_46_O_8_	4.02	557.3082	[M + Na]^+^	557.3085	−0.52	536.7906, 512.2362, 496.5966, 481.2945
				533.3131	[M-H]−	533.3109	4.13	427.2491, 515.3008, 497.2910, 301.1816
2	2,16,19,20,25-Pentahydroxycucurbita-5,23-diene-3,11,22-trione (2*α*,16*β*,20 *R*,23*E*)-form	C_30_H_44_O_8_	4.13	577.3022	[M + HCOOH-H]^−^	577.3007	2.56	425.2338, 513.2860. 495.2780, 443.2437
				555.2927	[M + Na]^+^	555.2928	−0.25	511.2225, 534.2230, 438.1933, 492.2522
3	2,16,20-Trihydroxycucurbita-1,5,25-triene-3,11,22-trione (16*α*,20 *R*)-form, 23,24*E*-didehydro, 2*-O-β*-D-glucopyranoside	C_36_H_50_O_11_	4.11	681.3244	[M + Na]^+^	681.3245	−0.20	327.1031, 349.1274, 563.2526
4	22-Deoxocucurbitacin D	C_30_H_46_O_6_	4.62	503.3363	[M + H]^+^	503.3367	−0.83	485.3256, 467.3154, 449.3045, 167.1062, 185.1172
5	22-Deoxocucurbitoside B	C_42_H_62_O_14_	4.82	813.4032	[M + Na]^+^	813.4032	0.03	569.7445, 699.3225, 319.2262, 495.1852
				835.4121	[M + HCOOH-H]^−^	835.4111	1.24	789.4066, 643.3511, 505.2947
6	25-Hydroxycucurbita-5,23-diene-3,7-dione	C_30_H_46_O_3_	8.43	477.3333	[M + Na]^+^	477.3339	−1.29	433.2312, 394.0185
7	3,7,23-Trihydroxycucurbita-5,24-dien-19-al (3*β*,7*β*,23 *S*)-form, 7*-O-β*-D-glucopyranoside	C_36_H_58_O_9_	7.13	657.3969	[M + Na]^+^	657.3973	−0.61	301.1422, 427.2155, 205.0293
				679.4064	[M+HCOOH-H]^−^	679.4052	1.78	633.4005, 471.3463, 356.7645
8	3,7,25,26-Tetrahydroxycucurbita-5,23-dien-19-al (3*β*,7*β*,23*E*,25*ξ*) form, 7*-O-β*-D-glucopyranoside	C_36_H_58_O_10_	4.15	673.3921	[M+Na]^+^	673.3922	−0.18	657.3561, 205.0273, 189.1658
				695.4009	[M+HCOOH-H]^−^	695.4001	1.14	649.3140, 605.5615, 493.3949
9	3,7,25-Trihydroxycucurbita-5,23-dien-19-al	C_30_H_48_O_4_	7.91	495.3448	[M+Na]^+^	495.3445	0.64	451.355
10	3,7,4′-Trihydroxyflavone (5-Deoxykaempferol)	C_15_H_10_O_5_	4.14	293.0423	[M+Na]^+^	293.0420	0.87	NP
				269.0452	[M-H]^−^	269.0445	2.79	NP
11	3-Hydroxy-7-methoxy-27-norcucurbita-5,23-dien-25-one	C_30_H_48_O_3_	3.96	457.3679	[M+H]^+^	457.3676	0.61	305.2477, 179.1431, 439.3565, 421.3459, 287.2360
12	6-*C*-*β*-D-Glucopyranosyl-4′,5,7-trihydroxyflavone 2”*-O-*[4-hydroxy-*E*-cinnamoyl-( → 6)-*β*-D-glucopyranosyl]	C_36_H_36_O_17_	3.37	741.2042	[M + H]^+^	741.2025	2.26	433.1130, 579.1417, 313.0706, 147.0440, 415.1021, 367.0809, 397.0913
				739.1885	[M-H]^−^	739.1869	2.20	593.1515, 413.0880, 293.0450, 265.0723, 341.0659
13	6-*C*-*β*-D-Glucopyranosyl-4′,5,7-trihydroxyflavone 2”*-O-*[4-hydroxy-3-methoxy-E-cinnamoyl-( → 6)-*β*-D-glucopyranosyl]	C_37_H_38_O_18_	3.37	771.2118	[M + H]^+^	771.2131	−1.67	433.1101, 415.1032, 177.0546, 313.0703, 367.0790, 337.0723
				769.1986	[M-H]^−^	769.1974	1.51	413.0882, 593.1479, 523.1353, 235.0612
14	6-Methoxyluteolin	C_16_H_12_O_7_	3.91	315.0510	[M-H]^−^	315.0499	3.40	301.0311, 271.0223, 255.0294,
15	Acutoside A	C_42_H_68_O_13_	6.20	803.4560	[M + Na]^+^	803.4552	0.98	641.4027, 191.1791, 439.3573, 349.0723
				825.4639	[M + HCOOH-H]^−^	825.4631	0.97	618.4083, 207.0506, 779.4564, 659.4155
16	Apigenin-7*-O-β*-D-glucopyranoside (Apigetrin)	C_21_H_20_O_10_	3.34	455.0948	[M + Na]^+^	455.0949	−0.15	437.0853, 365.0682, 335.0546
				431.0986	[M-H]^−^	431.0973	3.08	311.0560, 341.0665, 269.0479
17	Bryoamaride	C_36_H_54_O_12_	3.77	701.3502	[M + Na]^+^	701.3507	−0.78	349.1250
				723.3603	[M+HCOOH-H]^−^	723.3586	2.30	677.3542, 497.2909, 659.3444, 515.3020
18	Chrysoeriol	C_16_H_12_O_6_	4.28	299.0555	[M-H]^−^	299.0550	1.63	284.0320, 255.0291, 227.0371
19	Colocynthoside A	C_38_H_54_O_14_	3.53	757.3406	[M+Na]^+^	757.3406	0.03	697.3188, 365.1202
				779.3496	[M+HCOOH-H]^−^	779.3485	1.46	733.3436, 553.2810, 493.2604, 672.2780
20	Cucurbit-5-ene-3,23,24,25-tetraol (3*β*,23 *R*,24 *R*)-form 3*-O-*[*β*-D-galactopyranosyl-(1 → 6)-*β*-D-galactopyranoside]	C_42_H_72_O_14_	4.26	823.4818	[M + Na]^+^	823.4814	0.42	349.0710, 423.3627, 582.7825, 307.0598, 189.1628, 739.9498
				845.4902	[M+HCOOH-H]^−^	845.4893	1.05	799.4849, 637.4320
21	Cucurbit-5-ene-3,23,24,25-tetrol (3*β*,23 *R*,24 *S*)-form,3*-O-*[*β*-D-glucopyranosyl-(1 → 6)-*β*-D-glucopyranoside], 25*-O-β*-D-glucopyranoside	C_48_H_82_O_19_	3.84	985.5335	[M + Na]^+^	985.5343	−0.76	349.0692, 307.0597, 501.2505, 582.7740
22	Cucurbita-5(10),6,23-triene-3,25-diol 3*β*-form	C_30_H_48_O_2_	3.86	441.3722	[M + H]^+^	441.3727	−1.15	423.3641, 231.1363, 173.1346, 189.1617
23	Cucurbita-5,23-diene-3,7,25-triol (3*β*,7*β*,23*E*)-form	C_30_H_50_O_3_	4.26	441.3728	[M-H_2_O + H]^+^	441.3727	0.21	423.3618, 189.1643, 161.1326, 203.1793
24	Cucurbita-5,24-diene-3,7,22,23-tetrol (3*β*,7*α*,22 *S*,23 *S*)-form, 3,23-di*-O-β*-D-allopyranoside	C_42_H_70_O_14_	4.44	821.4653	[M + Na]^+^	821.4658	−0.58	349.0706, 581.2703, 247.0392, 499.2150, 419.2195
				843.4753	[M + HCOOH-H]^−^	843.4737	1.94	797.4697, 635.4164
25	Cucurbitacin A 2*-O-β*-D-glucopyranoside	C_38_H_56_O_14_	3.72	759.3564	[M + Na]^+^	759.3562	0.23	715.2998, 553.2460, 365.0908, 634.2750
26	Cucurbitacin C	C_32_H_48_O_8_	4.74	583.3236	[M + Na]^+^	583.3241	−0.92	523.3031, 567.2886, 437.7494, 541.3094
				605.3330	[M + HCOOH-H]^−^	605.3320	1.61	481.2963, 499.3059, 559.3277, 541.3163
27	Cucurbitacin D	C_30_H_44_O_7_	4.20	539.2972	[M + Na]^+^	539.2979	−1.34	342.9670, 181.0847
28	Cucurbitacin E	C_32_H_44_O_8_	4.84	579.2935	[M + Na]^+^	579.2928	1.14	519.2721, 485.2119, 355.1863
29	Cucurbitacin E 2*-O-β*-D-glucopyranoside	C_38_H_54_O_13_	4.08	741.3460	[M + Na]^+^	741.3457	0.46	681.3248, 597.7910, 349.1254
				763.3550	[M + HCOOH-H]^−^	763.3535	1.90	717.3487, 657.3276, 495.2745, 699.3376
30	Cucurbitacin F	C_30_H_46_O_7_	4.15	541.3136	[M + Na]^+^	541.3136	0.05	483.2726, 531.7472, 465.2681
				563.3226	[M + HCOOH-H]^−^	563.3215	2.02	517.3162, 499.3065, 385.2386
31	Cucurbitacin I	C_30_H_42_O_7_	3.65	515.3006	[M + H]^+^	515.3003	0.52	497.2895, 479.2755, 385.1999
32	Cucurbitacin I 2*-O-β*-D-glucopyranoside	C_36_H_52_O_12_	3.75	699.3352	[M + Na]^+^	699.3351	0.15	671.3258, 598.2973, 349.1256
				721.3439	[M + HCOOH-H]^−^	721.3430	1.27	675.3383, 657.3262, 497.2907, 341.1762, 513.2852
33	Cucurbitacin J 2*-O-β*-D-glucopyranoside	C_36_H_54_O_13_	3.65	717.3460	[M + Na]^+^	717.3457	0.47	633.8128, 349.1253, 497.2779
				739.3548	[M + HCOOH-H]^−^	739.3535	1.69	605.2963, 675.3383, 425.2335, 513.2860
34	Cucurbitacin P	C_30_H_48_O_7_	3.64	543.3294	[M + Na]^+^	543.3292	0.32	NP
				565.3382	[M + HCOOH-H]^−^	565.3371	1.93	519.3332, 501.3227, 471.3118, 357.2429, 489.3237
35	Cucurbitacin Q	C_32_H_48_O_8_	4.54	583.3233	[M + Na]^+^	583.3241	−1.44	523.303
				605.3329	[M + HCOOH-H]^−^	605.3320	1.45	559.3278, 481.2967, 499.3080, 541.3182
36	Cucurbitacin S	C_30_H_42_O_6_	3.76	499.3051	[M + H]^+^	499.3054	−0.63	481.2944, 317.2113, 385.2015, 463.2838
37	Cucurbitacin S 2*-O-β*-D-glucopyranoside	C_36_H_52_O_11_	3.77	683.3399	[M+Na]^+^	683.3402	−0.41	349.1261, 158.9628
38	Dihydrocucurbitacin C	C_32_H_50_O_8_	4.62	585.3393	[M+Na]^+^	585.3398	−0.84	525.3183, 225.0703, 485.3231, 183.0586
				607.3490	[M+HCOOH-H]^−^	607.3477	2.18	561.3439, 483.3123, 501.3229, 543.3337, 359.2238
39	Kaempferol 3*-O-*neohesperidoside	C_27_H_30_O_15_	3.27	595.1657	[M+H]^+^	595.1657	−0.08	433.1126, 415.1019, 313.0704, 337.0703, 367.0811, 271.0584
				593.1513	[M-H]^−^	593.1501	2.03	413.0876, 293.0464, 473.1101, 542.1834
40	Karavilagenin D	C_30_H_46_O_4_	6.87	493.3287	[M+Na]^+^	493.3288	−0.27	448.9751, 288.9226, 235.0106
41	Karaviloside IX	C_42_H_68_O_14_	5.33	819.4501	[M+Na]^+^	819.4501	−0.03	349.0690
42	Karaviloside XIII	C_36_H_58_O_8_	7.55	641.4013	[M+Na]^+^	641.4024	−1.70	479.3519, 560.9857. 512.9451, 185.0430
				663.4110	[M+HCOOH-H]^−^	663.4103	1.09	455.3527, 617.4050, 207.0499, 371.3616
43	Khekadaengoside K	C_30_H_42_O_10_	3.57	585.2668	[M+Na]^+^	585.2670	−0.37	349.1258, 423.2119, 501.7384
				561.2706	[M-H]^−^	561.2694	2.10	543.2603, 399.2198
44	Kuguacin F	C_30_H_42_O_5_	4.82	483.3109	[M+H]^+^	483.3105	0.83	341.2108, 441.2995, 383.2207
45	Kuguacin G	C_30_H_44_O_6_	4.53	501.3209	[M+H]^+^	501.3211	−0.33	483.3107, 465.2999, 447.2893, 327.2320, 285.1849
46	Kuguacin H	C_30_H_44_O_5_	6.29	507.3069	[M+Na]^+^	507.3081	−2.36	317.1040
47	Kuguaglycoside D	C_30_H_50_O_4_	6.55	497.3603	[M+Na]^+^	497.3601	0.34	NP
48	Luteolin 7*-O-β*-D-glucopyranoside (Cynaroside)	C_21_H_20_O_11_	3.20	449.1076	[M+H]^+^	449.1078	−0.53	299.0554, 353.0655, 329.0655, 395.0760, 413.0886
				447.0930	[M-H]^−^	447.0922	1.82	357.0625, 327.0507, 429.0831, 297.0386
49	Meloside A	C_36_H_36_O_18_	3.30	757.1970	[M+H]^+^	757.1974	−0.58	433.1122, 313.0704, 415.1018, 163.0388, 397.0901, 367.0813
				755.1831	[M-H]^−^	755.1818	1.73	593.1515, 413.0893, 281.0664, 341.0870, 179.0350
50	Momordicoside E	C_37_H_60_O_12_	4.25	695.4009	[M-H]^−^	695.4001	1.14	487.3411, 650.4022, 179.0559, 473.8553
51	Momordicoside O	C_42_H_68_O_15_	3.67	835.4450	[M+Na]^+^	835.4450	−0.05	674.3980

*NP = Not performed.

The identification of natural products was performed through the acquisition of full range mass spectra, and it was observed that most analytes, under the positive ionization source conditions, were observed as sodium adducts while a few were observed as protonated adducts. The formed sodium adducts were observed to be stable as they did not exhibit extensive fragmentation under CID conditions. Fragments with high abundances were generated through the loss of H_2_O or acetate group (if present), while other fragments were only seen in low abundances. In the negative ionization mode, mass spectra contained formate adducts, while deprotonated molecules ([M-H]^−^) were also seen. It was observed that the formate adducts, upon fragmentation, resulted in the loss of formic acid and generated deprotonated molecules which exhibited further fragmentation behaviour, such as the loss of water and acetate group.

**Figure 4:**
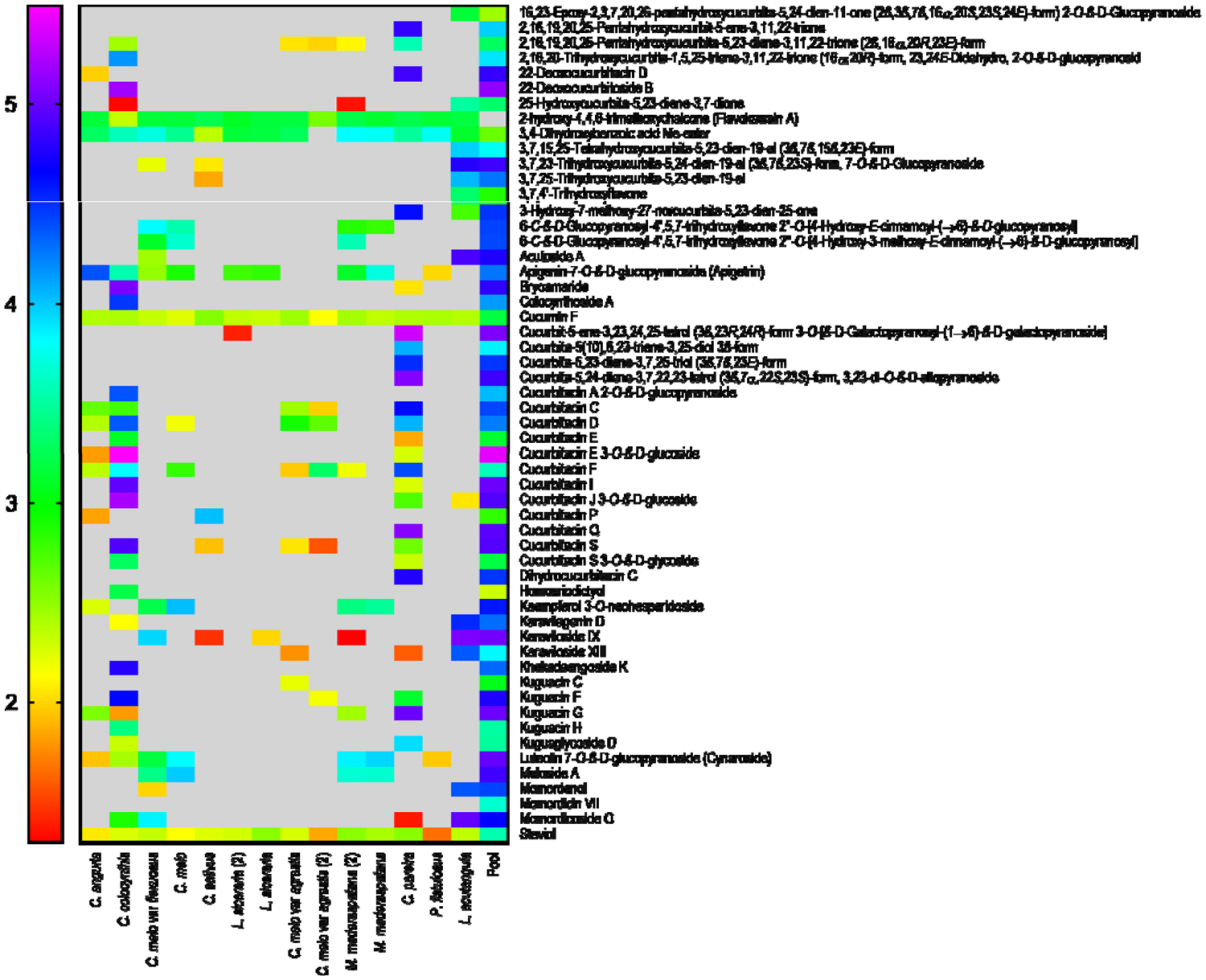
Heat map of compounds identified in positive ionization mode.

**Figure 5:**
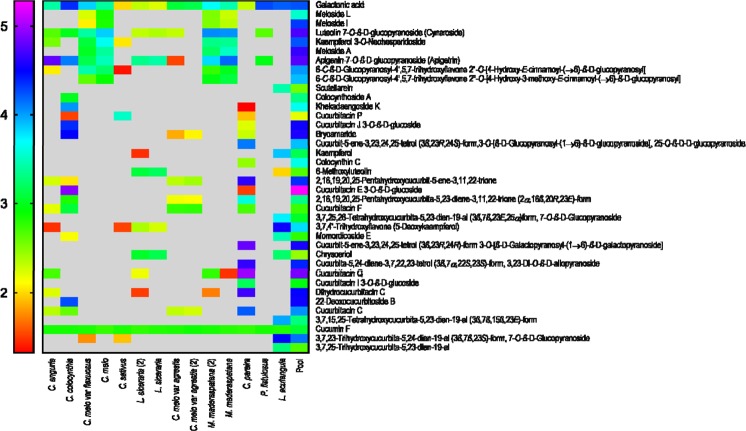
Heat map of compounds identified in negative ionization mode.

Based on the ion intensities observed, heat maps were generated for both ionization modes (positive and negative) to show a distribution of various plant metabolites across 6 genera and 10 species of the Cucurbitaceae family (Figs 4, 5). Heat maps were generated using GraphPad Prism 7 on a PC running Windows 7 SP1.

The developed quantitation method was applied for the detection and determination of analytes **1**–**4** in 10 different plants of the Cucurbitaceae family, along with *Cissampelos pareira* which belongs to the family Menispermaceae. This plant is rich in alkaloids and finds some uses in the Indian and Chinese medicine. Many alkaloids from this plant exhibit cytotoxic^23,30^, anti-inflammatory^20^, antiplasmodic activities and this is why it has gained some attention as a natural remedy for malaria in Kenya due to its antimalarial properties^31^. Although this plant is well-known for its alkaloidal content, our research group recently isolated cucurbitacins F and Q from this plant. The isolation of cucurbitacin F and Q is quite surprising from *C. pareira*. The structures of compounds were confirmed through ^1^H-NMR and ^13^C-NMR spectroscopy. The details of isolation for analytes **1**–**3**, along with ^1^H- and ^13^C-NMR data provided with supplementary information. Due to the isolation of analyte **3** from *C. pareira*, it was important to include this plant in the list of profiling of cucurbitacins. The results of quantitation showed that analytes **1**–**4** in various plants (in this study) occur in a very large range of concentrations between 0.12–5153.6 mg/Kg. The results of quantitation study are summarized in Table 3.

**Table 3:** Quantitation of analytes 1–4 in Cucurbitaceae plants.

Sample	Analyte conc. (mg/Kg of plant material)			
	1	2	3	4
*Citrullus colocynthis*	2.46 × 10^3^	5.15 × 10^3^	2.24 × 10^2^	2.89 × 10^3^
*Cucumis sativus*	8.40	7.31	0.270	10.8
*Cucumis melo*	3.65	4.00	0.12	5.48
*Cucumis melo var. flexuosus*	nd	3.94	nd	4.48
*Cucumis melo var. agrestis*	4.17	18.0	27.9	15.1
*Cucumis melo var. agrestis* (2)	nd	4.16	22.6	5.65
*Cucumis anguria*	3.22	3.90	38.2	5.54
*Luffa acutangula*	nd	4.34	0.78	5.20
*Lagenaria siceraria*	4.16	2.94	nd	5.79
*Lagenaria siceraria* (2)	8.10	7.10	nd	11.6
*Praecitrullus fistulosus*	nd	4.33	nd	4.96
*Praecitrullus fistulosus* (2)	nd	9.20	nd	11.2
*Mukia maderaspatana*	nd	0.74	15.5	6.13
*Mukia maderaspatana* (2)	nd	3.92	2.26	4.64
*Cissampelos pareira*	2.84	2.65	2.03 × 10^3^	5.62

*nd = not detected.

Analytes **1**–**4** were found to be present in significant quantities in *Citrullus colocynthis* which is well known for its cucurbitacin content. This plant exhibits various important bioactivities such as antidiabetic, anticancer, anti-inflammatory, etc.^32,33^. The fruits of *C. colocynthis* have been used traditionally in the Indo-Pak region for its antidiabetic properties^34^. The results of the current quantitation study concur with the traditional use *C. colocynthis* fruits as it contains high concentrations of cucurbitacins. Cucurbitacins E, I and Q have been shown to possess antitumor and antidiabetic activities^35–40^. *C. pareira* contains a substantial amount of cucurbitacin Q as found in our study and this concurs with the antiproliferative potential of the plant^30,41^. This does not signify that the anticancer potential of this plant is only due to the presence of a large amount of cucurbitacin Q as it requires further studies. Another interesting concurrence is the presence of analytes **1**–**4** in moderate amounts in other plants of the Cucurbitaceae family. These plants exhibit antidiabetic, anticancer, antibacterial, and other activities^42–44^.

## Conclusion

The present study has putatively identified fifty-one compounds in ten species in six different genera of the Cucurbitaceae family and *C. pareira* of the Menispermaceae family using high-resolution masses and fragmentation data. Mass spectrometric data of the identified compounds was used to produce a distribution profile these compounds in the analyzed Cucurbitaceae plants. A quantitation method for four bioactive cucurbitacins in the Cucurbitaceae plants was also developed in the current study. The developed quantitation method is simple, rapid, and sensitive. The results of this study are useful for natural product chemistry, food quality control, herbal products standardization, and drug discovery and development.

## Supplementary information

Metabolite Profiling and Quantitation of Cucurbitacins in Cucurbitaceae Plants by Liquid Chromatography coupled to Tandem Mass Spectrometry
