# Supplementary material for: Metabolite Profiling and Quantitation of Cucurbitacins in Cucurbitaceae Plants by Liquid Chromatography coupled to Tandem Mass Spectrometry
Source: Sci Rep. 2019 Nov 5;9:15992. doi: 10.1038/s41598-019-52404-1 (PMC6831693; doi:10.1038/s41598-019-52404-1)
Supplement: Supplementary file 1 — Metabolite Profiling and Quantitation of Cucurbitacins in Cucurbitaceae Plants by Liquid Chromatography coupled to Tandem Mass Spectrometry [file 41598_2019_52404_MOESM1_ESM.pdf]

Supplementary Information

Metabolite Profiling and Quantitation of Cucurbitacins in Cucurbitaceae Plants by Liquid Chromatography coupled to Tandem Mass Spectrometry

Faraz Ul Haq[a], Arslan Ali[b], Muhammad Noman Khan[a], Syed Muhammad Zaki Shah[a], Ram Chandra Kandel[c], Nudrat Aziz[a], Achyut Adhikari[c], M. Iqbal Choudhary[a,b,d], Atta-ur-Rahman[a,b], Hesham R. El-Seedi[e,f,*], and Syed Ghulam Musharraf[a,b,*]

[a]H.E.J. Research Institute of Chemistry, International Center for Chemical and Biological Sciences, University of Karachi, Karachi-75270, Pakistan

[b]Dr. Panjwani Center for Molecular Medicine and Drug Research, International Center for Chemical and Biological Sciences, University of Karachi, Karachi-75270, Pakistan

[c]Central Department of Chemistry, Tribhuvan University, Kirtipur, Kathmandu, Nepal

[d]Department of Biochemistry, Faculty of Science, King Abdulaziz University, Jeddah-21452, Saudi Arabia

[e]Pharmacognosoy Group, Department of Medicinal Chemistry, Uppsala University, Biomedical Centre, Box 574, SE-75 123, Uppsala, Sweden

[f]Alrayan Medical College, Medina 42541, Kingdom of Saudi Arabia

Correspondence

Prof. Dr. Syed Ghulam Musharraf and Dr. Hesham R. El-Seedi
E-mail address: musharraf1977@yahoo.com and hesham.el-seedi@fkog.uu.se

Method performances and validation

Ten calibrants for each analyte were used between the concentration range of 50 ng/mL to 2000 ng/mL. Linear calibration curves were obtained with correlation coefficients ≥ 0.996. Conservative fitting options were used to generate calibration curves with no weighing option or forced origin. LOD and LOQ values for the analyzed compounds were calculated using the standard deviation of the response (σ) and the slopes (S) i.e. LOD = 3.3σ/S and LOQ = 10σ/S. LOD and LOQ values were found to be between 0.64–0.90 ng/mL and 2.1–3.0 ng/mL, respectively. Supplementary Table 1 summarizes obtained LOD and LOQ values along with calibration equations. LOD and LOQ values indicate excellent sensitivity and selectivity of the developed method. Method accuracy and precision (intraday and interday precision) were calculated using three QC levels at 175, 875, and 1375 ng/mL, respectively. Accuracy was calculated as % bias while precision of the method was calculated in terms of % RSD. Accuracy of the method was found to be > 95% in all cases, while % RSD was found to be lower than 5% in all cases. The data for accuracy and bias of standard is listed in Supplementary Table 2.

For validation of analysis results, all plant samples were fortified with analytes **1**–**4** at three concentration levels: 50, 100 and 150 ng/mL. The fortified samples were marked as S1, S2, and S3 respectively, following the original sample name. Analyses of fortified samples showed increased concentrations of cucurbitacins in all samples and excellent recoveries in the range of 90–110% were observed. These recoveries show that the developed method is accurate and reproducible. The data from the recovery study is presented in Supplementary Table 3.

Supplementary Table 1: Summary of calibration parameters, limit-of-detection (LOD), and limit-of-quantitation (LOQ) for the optimized method.

Analyte	Regression Equation	R^2	LOD (ng/mL)	LOQ (ng/mL)
**1**	y = 10727.68x + 174281.54	0.999	0.90	3.0
**2**	y = 63410.53x + 1061811.2674281.54	0.999	0.64	2.1
**3**	y = 44321.60x + 75041.88	0.998	0.86	2.9
**4**	y = 15229.12x + 268803.32	0.996	0.67	2.2

Supplementary Table 2: Accuracy and precession data for analytes **1–4** analyzed at three QC levels.

Analyte	C_E (ng/mL)	Intra-day Analysis			Inter-day Analysis		
		C_M (ng/mL)	Precision (% RSD)	Accuracy (% Bias)	C_M (ng/mL)	Precision (% RSD)	Accuracy (% Bias)
**1**	175	182.5	2.90	4.29	184.3	3.15	5.31
	875	915.2	3.54	4.59	920.3	3.85	5.18
	1375	1408.9	2.38	2.47	1410.5	2.52	2.58
**2**	175	179.3	2.29	2.46	179.5	2.34	2.57
	875	906.3	2.92	3.58	915.2	3.11	4.59
	1375	1420.5	2.83	3.31	1430.4	2.76	4.03
**3**	175	168.2	3.69	-3.89	172.4	3.42	-1.49
	875	895.6	4.00	2.35	915.7	3.12	4.65
	1375	1350.6	3.35	-1.77	1365.1	3.53	-0.72
**4**	175	172.6	2.26	-1.37	182.4	2.30	4.23
	875	855.6	3.25	-2.22	901.4	3.14	3.02
	1375	1381.5	3.09	0.47	1400.7	3.13	1.87

Values are expressed as mean concentrations (n=6).

Supplementary Table 3: Determined recoveries of analytes **1**–**4** at three spike levels.

**Sample**	**Percent Recoveries**											
	**1**			**2**			**3**			**4**		
	**S1**	**S2**	**S3**	**S1**	**S2**	**S3**	**S1**	**S2**	**S3**	**S1**	**S2**	**S3**
Citrullus colocynthis	107.5	109.2	105.8	101.2	105.6	97.6	95.2	98.3	91.5	102.5	106.5	108.4
Cucumis sativus	106.5	104.2	103.5	107.4	103.3	104.8	108.6	101.1	95.2	96.3	98.4	100.8
Cucumis melo	106.2	108.3	104.6	102.5	108.6	109.4	104.5	93.5	92.8	99.4	104.6	108.7
Cucumis melo var. flexuosus	-	-	-	104.5	108.9	101.2	-	-	-	101.1	108.2	107.7
Cucumis melo var. agrestis	102.3	105.2	104.8	106.3	110.1	110.8	98.8	95.5	92.2	93.4	105.2	104.3
Cucumis melo var. agrestis (2)	-	-	-	108.7	106.4	109.2	109.3	104.4	108.7	108.9	99.5	99.2
Cucumis anguria	109.7	106.2	108.4	109.3	105.1	102.2	108.2	98.7	91.1	93.2	94.1	92.2
Luffa acutangula	-	-	-	96.2	108.2	109.3	107.3	102.2	101.5	109.5	106.5	102.2
Lagenaria siceraria	103.3	105.6	107.8	95.2	98.4	97.3	-	-	-	90.8	108.2	106.4
Lagenaria siceraria (2)	93.2	109.7	108.4	107.6	109.3	92.1	-	-	-	105.1	109.1	110.8
Praecitrullus fistulosus	-	-	-	107.2	108.5	108.1	-	-	-	100.5	108.2	102.6
Praecitrullus fistulosus (2)	-	-	-	102.5	103.4	105.3	-	-	-	109.1	100.2	100.9
Mukia maderaspatana	-	-	-	101.5	104.7	107.1	109.4	108.5	107.3	101.4	104.3	108.2
Mukia maderaspatana (2)	-	-	-	99.2	98.7	95.2	105.1	97.0	99.0	95.1	105.2	109.6
Cissampelos pareira	100.9	109.6	95.2	108.3	108.4	91.0	92.0	98.3	103.5	106.1	108.2	107.6

Isolation and purification of cucurbitacin E 2-*O*-*β*-D-glucopyranoside (1)

By examining results of TLC, the DCM fraction of methanolic extract of *Citrullus colocynthis* plant was loaded on a column packed with the silica gel and eluted with hexane to ethyl acetate solvents, which obtained impure sub-fractions. A sub-fraction was further passed through silica gel column, and eluted with 4% methanol in dichloromethane to isolate cucurbitacin E glycoside.

Supplementary Table 4: ^{13}C- and ^{1}H-NMR Chemical shift values of cucurbitacin E 2-*O*-*β*-D-glucopyranoside (**1**) (CD_3OD, 125, and 500 MHz, respectively).

C. No.	δ_C	δ_H (*J*, Hz)
1	123.7	6.09 d (2.0)
2	147.5	-
3	200.0	-
4	50.6	-
5	137.8	-
6	122.2	5.83 br s
7	25.03	2.38, 2.01 m
8	43.1	2.04 m
9	50.1	-
10	36.3	3.67 br s
11	216.4	-
12	49.9	(C-12a) 3.38 d (15.0), (C-12b) 2.60 d (15.0)
13	50.7	-
14	50.2	-
15	46.8	1.90 dd (12.7, 8.8), 1.47 br d (12.7)
16	72.1	4.56 t (8.0)
17	60.0	2.54 d (8.0)
18	20.7	1.00 s
19	20.5	1.28 s
20	80.1	-
21	25.6	1.40 s

22	205.1	-
23	122.7	6.83 d (16.0)
24	151.6	6.96 d (16.0)
25	80.8	-
26	26.9	1.56
27	26.9	1.56
28	21.1	0.89 s
29	28.4	1.25 s
30	19.9	1.29 s
1''	171.7	-
2''	22.0	2.0 s
1'	100.7	4.63 d (8.5)
2'	74.3	3.42
3'	77.6	3.40
4'	70.7	3.51
5'	78.2	3.33
6'	62.1	4.01 dd (12.0, 2.5), 3.84 (12.0, 4.5)

Isolation and purification of cucurbitacin I 2-*O*-*β*-D-glucopyranoside (2)

By examining results of TLC, the ethyl acetate fraction of methanolic extract of plant was subjected to silica gel column, eluted with methanol (8% and 2% respectively) in dichloromethane and obtained sub-fractions. A sub-fraction was purified over LH-20 sephadex by eluting with methanol resulting cucurbitacin I glycoside was isolate.

Supplementary Table 5: ^{13}C- and ^{1}H-NMR Chemical shift values of cucurbitacin I 2-*O*-*β*-D-glucopyranoside (**2**) (CD_3OD, 125, and 500 MHz, respectively).

C. No.	δ_C	δ_H (*J*, Hz)
1	123.2	6.09 d (2.0)
2	147.7	-
3	199.8	-
4	50.4	-
5	137.4	-

6	122.5	5.83 br s
7	24.7	2.41, 2.09 m
8	42.5	2.05 m
9	50.3	-
10	36.1	3.67 br s
11	216.2	-
12	49.7	(C-12a) 3.32 d (15.0), (C-12b) 3.26 d (15.0)
13	50.6	-
14	50.3	-
15	46.7	1.88 dd (12.7, 8.8), 1.47 br d (12.7)
16	71.9	4.56 t (8.0)
17	59.9	2.56 d (8.0)
18	20.9	0.88 s
19	20.3	1.28 s
20	80.0	-
21	27.1	1.49 s
22	205.3	-
23	123.0	6.83 d (16.0)
24	151.6	6.96 d (16.0)
25	71.6	-
26	26.0	1.56
27	26.0	1.56
28	21.4	0.99 s
29	28.0	1.24 s
30	18.3	1.40 s
1’	101.2	4.63 d (8.5)
2’	74.2	3.39
3’	77.7	3.40
4’	70.5	3.51
5’	78.2	3.33
6’	63.4	4.01 dd (12.0, 2.5), 3.84 (12.0, 4.5)

Isolation and purification of cucurbitacin Q (3) from *Cissampelos pareira*

The dried plants materials (1.2 kg) were chopped, pulverized and subsequently macerated in methanol for a period of 48 h at room temperature with daily shaking. After filtration the process was repeated three times using methanol (3 L) each time. The combined filtrates were concentrated in vacuum at 40º C to produce crude methanol extract (102 g, 8.5% w/w). Crude methanol was dissolved in distilled water and partitioned into *n*-hexane (10g), dichloromethane (15g), basic dichloromethane (12g) and ethyl acetate (3g) fractions. The dichloromethane fraction (16.06 g) was subjected to silica column chromatography and was eluted using ethyl acetate: hexane (1:99) with gradual increase of polarity to ethyl acetate (100 %) to yield sub fractions A-I. Out of them sub fraction E using 30% hexane/ ethyl acetate yielded cucurbitacin Q (**3**).

Supplementary Table 6: ^{13}C- and ^{1}H-NMR Chemical shift values of cucurbitacin Q (**3**) (CD_3OD, 125, and 500 MHz, respectively).

C. No.	δ_C	δ_H (J, Hz)
1	34.7	1.01m, 1.77 m
2	71.5	3.52 m
3	81.8	2.83 d (9.2)
4	42.1	-
5	142.0	-
6	119.9	5.73 br. d (5.6)
7	24.7	1.90, 1.97 m
8	44.3	1.91 m
9	49.0	-
10	34.8	2.37 m
11	216.4	-
12	46.6	1.39, 1.79 m
13	51.7	-
14	51.9	-
15	46.4	1.40, 1.81 m
16	71.6	4.52 t (8.0)
17	59.9	2.55 d (8.0)
18	20.5	0.84 s
19	20.6	1.05 s
20	81.9	-
21	25.2	1.38 s
22	205.1	-
23	122.7	6.81 d (16.0)

24	151.6	6.96 d (16.0)
25	80.8	-
26	26.9	1.53
27	26.9	1.55
28	22.3	0.95 s
29	25.6	1.16 s
30	19.7	1.29 s
31	171.8	-
32	21.9	1.99 s
